# A Review of the Neutrophil Extracellular Traps (NETs) from Cow, Sheep and Goat Models

**DOI:** 10.3390/ijms22158046

**Published:** 2021-07-28

**Authors:** Mulumebet Worku, Djaafar Rehrah, Hamid D. Ismail, Emmanuel Asiamah, Sarah Adjei-Fremah

**Affiliations:** 1 Department of Animal Sciences, North Carolina Agricultural and Technical State University, 1601 East Market Street, Greensboro, NC 27411, USA; drehrah@ncat.edu (D.R.); hdismail@ncat.edu (H.D.I.); 2 Department of Agriculture, University of Arkansas at Pine Bluff, 1200 N. University Drive, Mail Slot 4913, Pine Bluff, AR 71601, USA; asiamahe@uapb.edu; 3 Research & Economic Development, North Carolina Agricultural and Technical State University, 1601 East Market Street, Greensboro, NC 27411, USA; sadjeifr@ncat.edu

**Keywords:** neutrophil extracellular traps NETs, neutrophils, pathogens, humans, ruminants, health, therapy

## Abstract

This review provides insight into the importance of understanding NETosis in cows, sheep, and goats in light of the importance to their health, welfare and use as animal models. Neutrophils are essential to innate immunity, pathogen infection, and inflammatory diseases. The relevance of NETosis as a conserved innate immune response mechanism and the translational implications for public health are presented. Increased understanding of NETosis in ruminants will contribute to the prediction of pathologies and design of strategic interventions targeting NETs. This will help to control pathogens such as coronaviruses and inflammatory diseases such as mastitis that impact all mammals, including humans. Definition of unique attributes of NETosis in ruminants, in comparison to what has been observed in humans, has significant translational implications for one health and global food security, and thus warrants further study.

## 1. Introduction

The welfare and production of ruminant animals such as cows, sheep, and goats are impacted by pathogen induced and metabolic inflammatory diseases. Furthermore, species like cattle, sheep, and goats are useful model animals as they can be both target species for pathogens and reservoirs for human disease [1]. Neutrophils are granular leukocytes that are central to the inflammatory response. They are the most abundant innate immune cells, making up 50–70% of all leukocytes in humans [2], however, in ruminants such as cattle they comprise less than half of total circulating leukocytes [3,4]. Similarities have been reported in function of ruminant and human neutrophils [5]. Increased understanding of phenotypes and functions of neutrophils of different ruminant species will contribute to animal and public health.

Neutrophils are the first line of defense deploying sophisticated antimicrobial strategies [6] and also contributing to shaping adaptive immune responses [2,7]. The immune regulatory functions of neutrophils against pathogens include phagocytosis, release of antimicrobial molecules, production of reactive oxygen species (ROS), degranulation, and the formation of neutrophil extracellular traps (NETs), a process referred to as NETosis [8]. NETosis is a form of cell death, which is different from apoptosis and necrosis [9,10].

## 2. NETosis Mechanisms and Functions 

**Figure 1:**
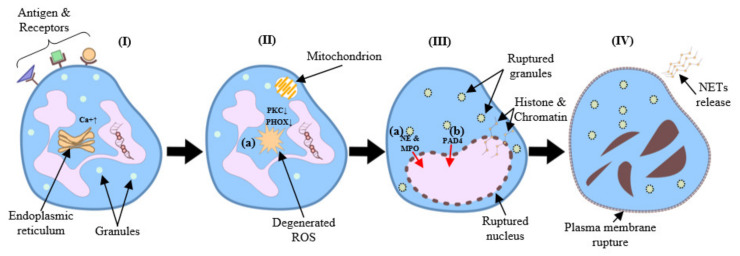
(**I**) Several stimuli (e.g., bacteria, viruses, fungi) initiate NETosis by binding to neutrophil receptors (e.g., Fc receptors, TLRs), which activate the endoplasmic reticulum to release stored calcium ions. (**II**) Elevated cytoplasmic calcium levels increase PKC activity, which induces NADPH oxidase to assemble into a functional complex (PHOX). (**II-a**) Subsequently, PHOX (or alternatively the mitochondrial respiratory chain) generate ROS. (**III**) ROS generation leads to the rupture of granules and the nuclear envelope. (**III-a**) Meanwhile, NE and MPO translocate to the nucleus. (**III-b**) As a result, histone deamination and chromatin de-condensation contribute to the formation of NETs. (**IV**) Finally, the rupture of the plasma membrane causes neutrophil lysis and allows the release of NETs.

NETs are large extracellular web-like structures [8] decorated with histones, antimicrobial proteins and DNA allowing them to trap and kill pathogens extracellularly [9,11,12,13,14] (Figure 1). When neutrophils undergo NETosis, nuclear and granular membranes disintegrate, the chromatin de-condenses, and it diffuses into the cytoplasm, mixing with cytoplasmic proteins [15]. Neutrophil components including neutrophil elastase (NE), myeloperoxidase (MPO), reactive oxygen species (ROS) [16], and peptidyl arginine deiminase 4 (PAD4) which citrullinates histones help to facilitate de-condensation and release of chromosomal DNA [6]. Both NADPH oxidase (NOX)-dependent and independent NETosis have been reported [17]. In addition to their role in host defense NETs are associated with pathologies acting as a double-edged sword in diseases [18,19,20]. As knowledge about NET’s increases, they are recognized as biomarkers of disease [21] for diagnosis and targeted therapy. Establishing efficient and accurate methods for quantifying NETosis under a variety of experimental conditions holds the potential to further elucidate the role of NETs and similar structures in normal and pathological processes [22].

## 3. Mammalian Neutrophil Extracellular Traps

### 3.1. Triggers and Phenotpes of Extracellular Traps

Neutrophils from different mammalian species including humans, horses, dogs, sheep, mice, as well as from invertebrates, form ETs [23,24,25,26,27,28,29,30,31,32,33,34,35,36]. In addition to neutrophils, ETs are formed by other immune cells such as mast cells, monocytes, macrophages, and eosinophils following stimulation with mitogens, cytokines, pathogens, or by interaction with neighboring cells and platelets [37]. Three different pathways result in ET formation: (1) release of nuclear DNA and cell death- suicidal cell pathway [12,38]; (2) release of nuclear DNA by viable cells—nonsuicidal vital NETosis [8,26,39,40,41,42]; and (3) release of mitochondrial DNA [43,44]. Genetic, species, and breed differences in mechanisms and efficiency of the NETosis response to pathogens have been reported [23,24,25] (Table 1).

Microbial pathogens that infect man and domestic animals can induce NETosis as part of the host’s innate immune response (Table 2) [8]. Diverse pathogen associated molecular patterns (PAMPS) such as the bacterial cell surface components LPS, lipoteichoic acid, and their breakdown products can trigger NETosis [36]. Formation of NETs can immobilize and kill microbes or inactivate microbial “virulence factors” and alter host cell function [9,16,40]. Microbes are able to circumvent NETosis using diverse mechanisms such as degrading NETs using nucleases [45]. Encapsulated pathogens or those that can change their surface charge to escape entrapment result in inflammation [46].

Formation of NETs contributes to the pathogenesis of inflammatory and autoimmune diseases in man. Pathologies associated with NET formation have been reported in systemic lupus erythematosus, rheumatoid arthritis [73], vasculitis [74,75], diabetes [76,77], atherosclerosis and cancer [78]. Excessive NET formation during sepsis [15,79], promotes thrombosis [80], thus enhancing coagulation and may also contribute to organ failure [81,82].

The purpose of this review is to present the current knowledge about the mechanisms of NETosis and its role in the pathogenesis of different diseases affecting three ruminants cows, sheep, and goats [8]. Further the aim is to present and discuss strategies to control parasites and inflammatory diseases by modulating NETosis through dietary or other interventions to promote animal welfare/health, product quality and translational efforts.

### 3.2. Extracellular Traps in Ruminants

Cattle, sheep, and goats are the major food-producing livestock worldwide [83]. Concerns regarding food insecurity are associated with inflammatory diseases and animal production [84,85]. The cost of parasitic disease is estimated at tens of billions of dollars worldwide [86]. Drug resistance impacts control of pathogens [87,88]. The innate immune system, primarily leukocytes, serves as the first line of host defense and plays a crucial role in early recognition and the proinflammatory response [89]. Understanding and interpreting neutrophil immune functions in different species [90] is essential to defining early defense mechanisms for better disease management.

Neutrophils are very important first line responders in inflammatory diseases which are associated with pathogen infection and metabolic disorders. They are central to the defense against pathogens causing disease such as mastitis, metritis, and parasitic infections as reviewed by Neumann et al., (2020) [36]. Impairments in neutrophil function such as during the periparturient period are associated with impaired animal health and welfare [91,92,93,94,95,96,97,98]. Furthermore, increased understanding of the regulation of neutrophil function is essential to the control of tissue damage resulting from cell activation and NETosis [99].

NETs have been characterized in several mammals including cows, sheep and goats, although not as extensively as in humans and mice [11]. Proinflammatory components of NET formation are associated with tissue damage in human lung [100] and in cow mammary epithelial cells [101]. It has been observed that direct proinflammatory effects on airway epithelial cells might contribute to recruitment of more neutrophils and perpetuation of inflammation, to cause lung tissue damage [101]. 

Understanding conserved and different responses of neutrophils from different species is essential to understanding host pathogen interactions to control diseases [90]. Comparative studies on NETosis are helping to advance knowledge about the role of neutrophils in reproduction to improve successful fertilization [102,103]. Understanding NETosis in ruminant species will aid in better definition of pathogenesis of diseases and designing of targeted therapeutics. Moreover, analyzing the responses of neutrophils of different species and NETs formation is relevant in light of zoonotic disease and the use of animal models for health [104,105,106]. Neutrophils are associated with lesions in the lungs and gastrointestinal tract of cows, sheep, and goats infected by coronaviruses and diverse pathogens [107]. Understanding NETosis in these species may help understand the pathophysiology and adaptability of animal and zoonotic pathogens while contributing to animal and public health through new targets for control [107].

#### NET Triggers in Cattle, Sheep and Goats

Cattle, sheep and goats are susceptible to inflammatory diseases caused by pathogens and as a result of metabolic disorders. These diseases are important limiting factors in production systems around the world resulting in economic losses [108]. The mechanism of activation, migration into tissue and immune modulation in response to stimuli, microbial killing and NETosis of cow, sheep and goat neutrophils is similar to other species [109].

In cows, stimulation of neutrophils with bacteria common to mammary gland infections leads to neutrophil extracellular traps formation in milk [27]. Studies in sheep have shown that NETosis is associated with changes in proteins such as TLR in response to pathogens that cause mastitis [72]. Pisanu et al., (2015) described NET formation in vivo where milk and tissues collected from the mammary gland of sheep that developed acute mastitis after experimental *Streptococcus uberis* infection, demonstrated the presence of extra nuclear DNA co-localizing with antimicrobial proteins, histones, and bacteria [72]. Histone citrullination formation plays a role in NETs found in mammary alveoli in response to *S. uberis* infection [72]. Studies on bovine mammary epithelial cells have implicated NET formation and in particular histones to be involved in mammary epithelial cell damage in vitro [101]. Targeted inhibition of excess NET formation may aid in combatting tissue damage [101,110]. In this study NET markers were markedly increased, 1095 unique proteins were identified, with 287 being significantly more abundant in mastitic milk [72]. These markers may aid in targeted inhibition of excess NET formation [72,110].

Cacciotto et al., (2016) described NET formation in vivo in the mammary gland and milk of sheep naturally infected by *Mycoplasma agalactiae* [111]. Sheep neutrophils formed NETs through binding of the lipoprotein to TLR2. Furthermore, the authors suggested that *M. agalactiae* may circumvent NETosis by degrading the DNA component of NETs through its surface nuclease MAG_5040. Thus promoting its survival and the establishment of persistent infections [111]. Understanding of microbial virulence factors may aid in design of novel diagnostics and therapeutics for the control of pathogens such as mycoplasma. Pathogens such as *Mycoplasma bovis*, can escape NET-mediated killing [57,58]. Relative senescence of individual cow neutrophils was associated with increased NET formation in response to repeated exposure to *M. haemolytica* [112]. Viable and heat-killed *M. tuberculosis* bacteria and unilamellar liposomes, as well as *Mycobacterium bovis* BCG were efficient NET inducers [51]. Although bacteria remained viable it was postulated that in vivo, neutrophils might propitiate recruitment and activation of more efficient microbicidal cells [51]. In addition to their direct interactions with invading pathogens, NETs can exert a direct enhancement or dampening effect on the inflammatory responses [11]. 

Neutrophils release ETs as a defense strategy against pathogens [24,27,28,31,32,65,67,68,70,113,114,115]. These parasites include *Eimeria bovis* [31,32,67], *Neospora caninum* [59], trypomastigotes [116] and *Haemonchus contortus* [64]. Pathogens can be entrapped and killed within NET-like structures in a ROS-dependent or independent manner [32,59,63]. The extracellular, haemoflagellate parasite *Trypanosoma brucei* is a cause of trypanosomiasis resulting in mortality and morbidity in cattle, sheep, goats, and horses. The entrapment of trypmastigotes in aggregated NETs was purinergic-dependent and maybe important in trypanosomiasis-related immune-pathological disorders [116]. *Neospora caninum* is an apicomplexan intracellular parasite of cattle and dogs that also cause clinical infections in horses, goats, sheep, and deer. It causes severe reproductive disorders in cattle worldwide. Neospora caninum, induced classical mammalian NET formation in cow and goat neutrophils [117,118]. *N.caninum*-induced NETosis appears to be influenced by MPO and CD11b, but independent of NADPH oxidase, store-operated calcium entry, ERK1/2 and p38 MAPK activities [117,118]. *Toxoplasma gondii*, a protozoan parasite that causes toxoplasmosis in warm-blooded animals triggers NETs in human, mouse, sheep and cattle neutrophils [68]. *T. gondii*-induced NETosis was dependent on tachyzoite concentrations and incubation time in both sheep and cattle. NETs structures released from sheep neutrophils caused mechanical immobilization of *T. gondii* tachyzoites. NETs structures and MPO may have a lethal effect on *T. gondii* tachyzoites in vitro [36,68]. Yildiz et al., (2017) reported that NET structures produced by sheep neutrophils may only ensnare *T. gondii* tachyzoites, whereas cattle neutrophils had lethal effects in vitro [68]. 

Cattle sheep and goats are infected with different species of the genus *Eimeria* a protozoan parasite causing coccidiosis. *Eimeria bovis* in cattle or *Eimeria arloingi* in goats are associated with health problems and economic losses especially in young animals [63,64,65]. Eimeria arloingi triggered the release of ROS-dependent caprine neutrophil ET fibers and were entrapped within the meshwork [33]. Although E. arloingi were immobilized within the NETs, this did not affect the viability of the parasites [33]. Findings from several studies reported that triggering of NETs is dependent on incubation time in bovine and caprine neutrophils against parasites such as E. bovis, C. parvum and E. arloingi sporozoites [32,33,65] contradicting what was reported with tachyzoites of B. besnoiti [34]. The induction of caprine NETs by E. arloingi was confirmed by Munoz-Caro et al., (2016), who reported colocalization of extracellular DNA with neutrophil elastase and histones in Eimeria-infected tissue samples [66]. Citrullinated histone H3, a typical NET marker for human and mouse NETs [119], was found in close proximity to Eimeria in different stages of replication. NADPH-oxidase-dependent NETosis was described in response to viable sporozoites, sporocysts, and oocysts of Eimeria ninakohlyakimovae, in association with increased IL-12 and TNFα in goats [61,62]. The authors hypothesized that the released DNA structures immobilized rather than killed the parasites. Moreover, caprine monocytes also released ETs [61,62].

Many species of parasitic helminths are impacted by NETosis as part of the early immune response of the host. The abomasal parasite *Haemonchus contortus* is a gastrointestinal nematode with worldwide distribution causing significant economic losses particularly in small ruminants. In cow and sheep neutrophils L3 larvae of *Haemonchus contortus* [64] induce different phenotypes such as aggregated NETs, spread NETs and diffuse NETs. Both disseminated and aggregated NETs entrapped L3 [64]. The viability of *H. contortus* was not affected by entrapment [68]. Studies have demonstrated that cow neutrophils release NETs in response to the free-living soil nematode *Caenorhabditis elegans*, that NET production may be a conserved mechanism against a broad range of nematodes. The cattle stomach worm *O. ostertagi*-induced NET formation by a ROS-independent and NADPH oxidase dependent pathway [120]. Fascioliasis is a zoonotic disease caused by infection with the trematode *Fasciola hepatica*, resulting in hepatitis in humans and livestock. Pathogens employ virulence factors and molecular mimicry to avoid detection or trigger immune modulatory factors to impact the NETotic response [121]. *Fasciola hepatica* secretes parasite-specific molecules to either resolve NETs or to impair NETosis signaling pathways to possibly impact disease pathology in vivo [122].Thus more studies are needed on pathogen recognition to shed light on mechanisms of immune evasion related to NETosis.

Exposure to molecular stimuli other than pathogens is also associated with the NET formation. For example, PMA, ionomycin or milk each induced NETosis in contrast to inhibition of phagocytosis and oxidative burst observed in cow blood [27]. The release of NETs, inhibition by milk components, and association of relevant proteins with the milk fat has been reported [27]. Beta-hydroxybutyrate, produced during ketosis or hyperketonemia, reduced phagocytosis and NET-mediated killing of *E. coli* P4 by neutrophils [28]. Alarcon et al., (2020) reported that nonesterified fatty acids (NEFAs), by inducing NET formation may contribute to postpartum diseases in cows. The effect of NEFA in cow neutrophils was faster than reports for human neutrophils [123]. Alacron et al., 2017 [60]. Reported that activation of NETosis with d (-) lactic may contribute to neutrophil-derived proinflammatory processes, such as aseptic laminitis and/or polysynovitis in animals suffering from acute ruminal acidosis [60]. Histamine regulates the immune response in allergic diseases such as asthma, rhinitis in man and laminitis in cows through regulating immune responses. Histamine-triggered NETosis increased ERK and p38s proteins and activation of NADPH oxidase in cows [124]. Thus changes in the host microenvironment that impact NETosis can impact disease outcome.

## 4. Prediction, Modulation and Therapy

The innate immune response involves evolutionarily conserved pathogen recognition receptors (PRR) that recognize PAMPS. This is exemplified by toll-like receptors (TLR), which recognize specific PAMPS such as LPS [125]. In light of reports of the role of TLR in NETosis, understanding the mechanisms underlying regulation of the inflammatory response to PAMPs in diseases such as mastitis and metritis may aid in the design of tailored therapeutics that target pathogen recognition and inflammation [126]. For effective use of NETs as biomarkers and targets for therapy, continued efforts are needed to identify and define the function of genes involved in innate immunity. Furthermore, we need to develop tools that identify and predict NETosis phenotypes in cattle, sheep and goats. Anti-NET therapeutics that target induction or inhibition of NET formation are being studied in human neutrophils [106]. The machine learning algorithm called convolutional neural network was used to quantitate and identify NETotic and non-NETotic classes with an accuracy of greater than 94% [127]. The design of new and implementation of improved machine learning tools may help capture unique attributes/features of NETosis in ruminants in comparison to what has been observed in human for increased understanding [127].

Development of novel anti-NET therapeutic strategies might help to reduce disease and improve animal welfare and production. Dietary modulation that targets NETosis may enhance the functional benefits while regulating harmful consequences [123]. Diverse stimuli are associated with differential and temporal modulation of gene expression in ruminant blood and in immune cells such as neutrophils [92,99,106].

Phytochemicals and probiotics [99,105,128,129] are being studied to augment innate immunity against microbes and gastrointestinal parasites to address concerns regarding antibiotic resistance. These include extracts such as: garlic, neem, wormwood, tobacco, cowpea [87,130,131] and *Sericea lespedeza* [132]. [Table 3]. As reported by Vong et al., (2014) probiotic *bacteria* inhibit both PMA- and *S. aureus*-induced NETosis [110]. Furthermore, this inhibition of NETosis is an additional benefit of PAMPS expressed by health beneficial microorganism playing a role in maintaining homeostasis and gastrointestinal health [95,108]. Genes associated with innate and adaptive immunity are differentially regulated in cows and goats receiving probiotics [95,108]. Adjei-Fremah et al., (2016) investigated the in vitro effect of LPS using blood samples collected from probiotics-treated animals. Global gene expression analysis identified 13,658 differentially expressed genes (fold change cutoff ≥ 2, *p* < 0.05), 3816 upregulated genes and 9842 downregulated genes in blood in response to LPS [133,134]. The regulation of the genes involved in inflammation signaling pathway suggests that probiotics may stimulate the innate immune response of animal against parasitic and bacterial infections [95] The effect of probiotics on NETosis in ruminants needs investigation.

Antioxidants can inhibit ROS-associated NET release [135,136]. Studies are needed to evaluate the effect of antioxidants as modulators of NETosis in cows, sheep and goats [110,131,133,134]. Polyphenolic extracts from cowpea (*Vigna unguiculata*) [98] have been shown to change gene expression [137] and activate signaling pathways such as the toll-like receptor pathway, inflammation response pathway and MAPK cascade pathways, among others [130,138]. Animal feed rich in phenolic constituents has immuno-regulatory effects in ruminants [139,140]. Forages rich in polyphenol such as *Sericea lespedeza* and cowpea also changed gene expression in goat blood [128,130,132,141]. Asiamah et al., [92] showed extracts from *Sericea lespedeza* modulated the expression of innate and adaptive immune and WNT signaling pathway genes including TLR2, TLR4, WNT5A and FZD. Table 3 shows genes, modulated as a result of probiotic and phytochemicals supplementation in goats [108,142], that may impact NETosis.

Dietary modulation to improve immuno-suppression during the periparturient period can impact NETosis and immune function [123] Global gene expression profiles of blood-derived neutrophils from periparturient cows revealed that 249 genes out of 44,000 were differentially expressed (fold change ≥ 2, *p* < 0.05). Eighty-seven (87) genes were down-regulated and among the top 20 downregulated genes were genes essential to neutrophil response and immunity [145], such as PGLYRP1 and SERPINB4. The observed downregulation of adhesion genes could lead to impaired NETosis during the periparturient period. Erpenbeck et al., (2019) reported a correlation between neutrophil adhesion/contact area and NETosis [99]. They demonstrated that, PMA induced NETosis is independent of adhesion and LPS-induced NETosis was dependent on adhesion to specific surfaces in the body [99].

Changes in neutrophil gene expression and function due to supplementation of the cow’s diet with rumen-protected methionine (Met) aid in alleviating immune suppression during the periparturient period [146,147,148,149]. A study by Stella et al., (2018) revealed that NET formation does not appear to be affected by limited amino acids during the periparturient period [150]. In vitro, addition of Met, as a scavenger of *hypochlorous* acid, a product of MPO, had no effect following stimulation with PMA or bacteria [151]. Methionine sulfoxidation is a post-translational modification observed in PMA- and LPS-induced NETs [152]. In cows Asiamah et al., (2019) reported an association between dietary Met supplementation and galectin gene expression and secretion [153].

Galectins are soluble β-galactoside-binding lectins that regulate immune function [154]. Galectin-9 is reported to co-localize with corpses of neutrophils following NETosis, suggesting a potential role in the clearance of neutrophils [155]. Supplementation of dairy cows with Met reduced the galectins involved in inflammation (galectins 1, 2, 3, 4 and 12) [143]. The increase of galectin 8 to Met supplementation in the presence of LPS in vitro, however, shows a possible pro-inflammatory role of this galectin in cows [156]. Furthermore, Met supplementation may improve neutrophil migration and phagocytic capacity in part by increasing the expression of galectin 8 in cow neutrophils [91,93]. Galectin 8 is involved in activating anti-bacterial autophagy [157]. Nishi et al., (2003) reported that galectin 8 induces a firm and reversible adhesion of peripheral blood neutrophils in vitro [156], which suggests that they play a vital role in neutrophil migration. It is possible that cows with more Met may respond better to infection through enhancement of adhesion in response to LPS and formation of NETs [153].

Breed and species-specific patterns of NETosis offer an opportunity to harness genomic technologies for targeted intervention and immunomodulation of conserved receptor signaling pathways using ruminants as models [125,158]. Studies are showing that NETs induced in different conditions may have different biological effects [152]. Using proteome analysis, it was shown that NETs induced by different stimuli had heterogeneous protein composition and post-translational modifications [152]. Changing the proteome by degrading NETs using deoxyribonuclease to reduce inflammation is a therapeutic approach with implications in both human and animal diseases [151]. In cattle sheep and goats, changes in NET markers and the proteome are associated with disease of concern to animal health and production [72]. Increased definition of the proteomes, associated post-translational modifications, and markers of NETosis in sheep cattle and goats will contribute to the development of therapeutics for inhibition of excess NETs formation to ameliorate pathologies associated with diseases [101,129,152,158].

## 5. Conclusions

In cattle, sheep, and goats NETosis is associated with the response to diverse zoonotic pathogens and those impacting animal health and welfare. Pathophysiological damage has been reported from NETosis in humans and ruminants. Increased understanding of NETosis in ruminants will help in interventions to control diseases such as mastitis that impact all mammals, including humans. Species specific responses to pathogens such as coronaviruses in relation to NET formation in ruminants, may contribute to definition of the role of neutrophils in pathophysiology and severity of diseases such as COVID-19. Studies on the innate immune response to pathogens and their modulation using natural products can serve as a springboard for definition of NETosis signatures and their modulation in ruminants. Furthermore, NETosis is associated with impaired fertilization, periparturient health, and diet. Improvements in methodology such as application of machine learning tools are essential to decipher and harness the components of the double edge sword of NET formation as biomarkers for disease and as targets for therapeutic intervention. Studies on the innate immune response to pathogens and their modulation using natural products can serve as a springboard for application of anti-NETosis therapeutics for cows, sheep and goats. Breed and species-specific patterns of NETosis offer an opportunity to harness genomic technologies for targeted intervention and immunomodulation of conserved receptor signaling pathways using ruminants as models. Definition of the NET proteome and its unique attributes/features in ruminants in comparison to what has been observed in humans has significant implications for the design of therapeutics for health and global food security, and thus warrants further study.

## Figures and Tables

**Table 1:** Summary of mechanisms of neutrophil extracellular trap formation in different species.

Microorganisms	Species	Mechanism	Types of NETosis	References
BACTERIA				
*Staphylococcus aureus*	Mice Humans Bovine	Dependent on TLR2 and Complement C3 in mice PAD4 dependent Response to virulence factor, PVL in a ROS independent manner DNA extruded via vesicles Unknown	Vital Vital Unknown	[26] [40] [27]
*E. coli*	Humans * Mice * Bovine	* Mediated via platelet TLR4 Histone H3 citrullination by PAD4	Vital in the presence of platelets	[39] [28]
*E. coli LPS*	Humans *	* Mediated via platelet TLR4 and present HMGB1 to neutrophils	Vital in vivo	[29]
**VIRUS**				
*Influenza A* *Influenza H1N1*	Mice Humans	Not dependent on PAD4 ROS and PAD4 dependent	Suicidal Suicidal	[30]
**PARASITES**				
*Eimeria bovis*	Bovine	Recognition by CD11b Dependent on NAPDH oxidase, NE and MPO Requires p38 MAPK and ERK1/2 phosphorylation	Unknown	[31] [32]
*Eimeria arloingi*	Goat	NADPH oxidase dependent	Unknown	[33]
*Besnoitia besnoiti*	Bovine	Dependent on NAPDH oxidase, NE and MPO	Unknown	[34]
*Toxoplasma gondii*	Humans Mice	ERK-MEK dependent * NADPH oxidase/ROS dependent	Suicidal	[35]

TLR, toll-like receptor; C3, complement 3; PAD4, peptidyl arginine deiminase 4; PVL, Panto-Valentine leukocidin; HMGB1, high mobility group box 1; ROS, reactive oxygen species; NE, neutrophil elastase; MPO, myeloperoxidase; MAPK, mitogen-activated protein kinase, ERK1/2; extracellular signal regulated kinase ½, NADPH; nicotinamide adenine dinucleotide phosphate. * Mechanism found in specified species.

**Table 2:** Microbial inducers of NETosis.

Inducer Type	Reference
*Staphylococcus aureus*	[40,47]
*Streptococcus sp.*	[48]
*Haemophilus influenzae*	[49]
*Klebsiella pneumoniae*	[16]
*Listeria monocytogenes*	[50]
*Mycobacterium tuberculosis*	[51]
*Shigella flexneri*	[9]
*Aspergillus nidulans*	[52,53,54]
*Aspergillus fumigatus*	
*Candida albicans*	
*Porphyromonas gingivalis*	[55]
*V. cholera*	[45]
*Aeromonas hydrophila*	[56]
*E. arloingi sporozoites, B. besnoiti, C. parvum, Spermatozoa, H. contortus, N. caninum, D (-) lactic acid, M. bovis, E. ninakohlyakimovae, T. gondii, S. uberis*	[24,31,32,33,34,57,58,59,60,61,62,63,64,65,66,67,68,69,70,71,72]

**Table 3:** Selected immunomodulators tested in goat blood.

Modulator (s)	Sample Type (s)	Cytokines	Innate Immune Response	Reference
Probiotics	Whole blood, serum	IL2, IL5, IL10, IL8, IL18	TLR4, TLR6, TLR7, TLR9	[96,143]
Cowpea	Whole blood, serum, plasma	TNFα, IL1α, ILβ, IL8 IL10RA, IL15, IP10, GCSF, Rantes and IFNγ	TLR2	[139]
Sericea lespedeza	Whole blood, Serum	TNF-α, IFNr, GCSF, GMCSF, IL-1α, IP-10	TLR2 and TLR4	[132]
Mushroom	Neutrophils, Whole blood, serum	IFNr, Rantes, GCSF, and GM-CSF	TLR1, TLR2, TLR3, TLR4, TLR5, TLR6, TLR7, TLR8, TLR9, TLR10	[142]
Lipopolysaccharide	Mammary epithelial cells, whole blood, blood leukocytes	IL1B, CCL3 and IL8, CCL2, CXCL6, IL6, CXCL8	PTGS2, IFIT3, MYD88, NFKB1, and TLR4	[92,144]

IL2, interleukin 2; TLR, Toll-like receptor; TNFα, tumor necrosis factor α; GCSF, granulocyte colony stimulating factor; IFNγ, interferon γ; GMCSF, granulocyte macrophage colony stimulating factor; IP-10, IFN-γ-induced protein 10; IFNr, interferon regulator; CCL3, macrophage inflammatory protein-1 α, MIP-1α; CXCL6, chemokine (C-X-C motif) ligand 6.

## Abbreviations

IFN	interferon
IP-10	IFN-γ-induced protein 10
IFN-α	interferon α
IL	interleukin
MPO	myeloperoxidase
NE	neutrophil elastase
ETs	extracellular traps
NETs	neutrophil extracellular traps
Nox	NADPH-oxidase
PMA	phorbol-12-myristate-13-acetate
ROS	reactive oxygen species
SARS-CoV-2	severe acute respiratory syndrome coronavirus 2
SLE	systemic lupus erythematosus
TNF-α	tumor necrosis factor-α
BPI	bactericidal permeability-increasing factor
ERK1/2	extracellular signal-regulated kinases 1/2
LPS	lipopolysaccharide
TLR	Toll-like receptor
C3	complement 3
PAD4	peptidyl arginine deiminase 4
PVL	Panto-Valentine leukocidin
HMGB1	high-mobility group box 1
MAPK	mitogen-activated protein kinase hi
NADPH	nicotinamide adenine dinucleotide phosphate
PAMPs	pathogen-associated molecular patterns
CD 11b	cluster of differentiation molecule 11b
CD14	cluster of differentiation antigen 14
PMNs	polymorphonuclear leukocytes
CH3	citrullinated Histone H3
NEFAs	nonesterified fatty acids
ICAM-1	intercellular Adhesion Molecule 1
PVD	purulent vaginal discharge
MAC-1	macrophage-1 antigen
FC	fold change
NFkB	nuclear factor kappa-light-chain-enhancer of activated B cells
IL1B	interleukin 1 beta or lymphocyte activating factor (cytokine protein)
FZD	frizzled
PGLYRP1	gene peptidoglycan recognition protein 1 gene
SERPINB4	protein serpin family B member 4 protein
PMNL	polymorphonuclear leukocytes
Met	methionine
RPM	rumen-protected Met
GCSF	granulocyte colony stimulating factor
GMCSF	granulocyte macrophage colony stimulating factor
CoV	coronavirus
BCoV	bovine CoV
PRRs	pattern recognition receptors
CNN	convolutional neural network
NEFAs	nonesterified fatty acids
CCL3	macrophage inflammatory protein-1 α, MIP-1α
CXCL6	chemokine (C-X-C motif) ligand 6
SOCE	store-operated calcium entry
